# Supplementary material for: Resolving the full spectrum of human genome variation using Linked-Reads
Source: Genome Res. 2019 Apr;29(4):635–45. doi: 10.1101/gr.234443.118 (PMC6442396; doi:10.1101/gr.234443.118)
Supplement: Supplemental Material [file supp_gr.234443.118_Supplemental_longranger-2.2.zip › longranger-2.2.tar.bz2!/lr2.2-public/lib/stan/cmdstan/stan/doc/api/html/functions_func_i.html]

Stan: Class Members - Functions


|  |  |
| --- | --- |
|  | Stan  2.5.0  probability, sampling & optimization |


- Main Page
- Related Pages
- Namespaces
- Classes
- Files

- Class List
- Class Index
- Class Hierarchy
- Class Members

- All
- Functions
- Variables
- Typedefs
- Enumerator
- Related Functions

- a
- b
- c
- d
- e
- f
- g
- h
- i
- j
- k
- l
- m
- n
- o
- p
- q
- r
- s
- t
- u
- v
- w
- x
- z
- ~


All Classes Namespaces Files Functions Variables Typedefs Enumerations Enumerator Friends Macros Pages

### - i -

- identifier\_exists()
  : validate\_identifier
- incr\_dim\_size()
  : stan::json::json\_data\_handler
- increment\_log\_prob\_statement()
  : stan::gm::increment\_log\_prob\_statement
- index()
  : stan::mcmc::chains< RNG >
- index\_op()
  : stan::gm::index\_op
- infer\_type()
  : stan::gm::fun
  , stan::gm::index\_op
- infinity()
  : std::numeric\_limits< stan::agrad::fvar< T > >
  , std::numeric\_limits< stan::agrad::var >
- init()
  : stan::mcmc::base\_hamiltonian< M, P, BaseRNG >
- init\_dependent()
  : stan::agrad::chainable
  , stan::agrad::vari
- init\_local\_var\_visgen()
  : stan::gm::init\_local\_var\_visgen
- init\_stepsize()
  : stan::mcmc::base\_hmc< M, P, H, I, BaseRNG >
- initial\_state()
  : stan::math::coupled\_ode\_system< F, double, double >
  , stan::math::coupled\_ode\_system< F, double, stan::agrad::var >
  , stan::math::coupled\_ode\_system< F, stan::agrad::var, double >
  , stan::math::coupled\_ode\_system< F, stan::agrad::var, stan::agrad::var >
- initialize()
  : stan::optimization::BFGSLineSearch< M, QNUpdateType, Scalar, DimAtCompile >
  , stan::optimization::BFGSMinimizer< FunctorType, QNUpdateType, Scalar, DimAtCompile >
- instance()
  : stan::gm::function\_signatures
- int\_literal()
  : stan::gm::int\_literal
- int\_values()
  : stan::io::dump\_reader
- int\_var\_decl()
  : stan::gm::int\_var\_decl
- integer()
  : stan::io::reader< T >
  , stan::io::writer< T >
- integer\_constrain()
  : stan::io::reader< T >
- integer\_lb()
  : stan::io::reader< T >
- integer\_lb\_constrain()
  : stan::io::reader< T >
- integer\_lub()
  : stan::io::reader< T >
- integer\_lub\_constrain()
  : stan::io::reader< T >
- integer\_ub()
  : stan::io::reader< T >
- integer\_ub\_constrain()
  : stan::io::reader< T >
- integrate\_ode()
  : stan::gm::integrate\_ode
- inverse()
  : stan::math::LDLT\_factor< T, R, C >
- is\_default()
  : stan::gm::list\_argument
  , stan::gm::singleton\_argument< T >
  , stan::gm::valued\_argument
- is\_defined()
  : stan::gm::function\_signatures
- is\_double\_return()
  : stan::gm::validate\_sample
- is\_ill\_formed()
  : stan::gm::expr\_type
  , stan::gm::sample
- is\_int()
  : stan::io::dump\_reader
- is\_no\_op\_statement()
  : stan::gm::statement
- is\_present()
  : stan::gm::unvalued\_argument
- is\_primitive()
  : stan::gm::expr\_type
- is\_primitive\_double()
  : stan::gm::expr\_type
- is\_primitive\_int()
  : stan::gm::expr\_type
- is\_recording()
  : stan::common::recorder::csv
  , stan::common::recorder::filtered\_values< InternalVector >
  , stan::common::recorder::messages
  , stan::common::recorder::no\_op
  , stan::common::recorder::sum\_values
  , stan::common::recorder::values< InternalVector >
- is\_uninitialized()
  : stan::agrad::var
- is\_univariate()
  : stan::gm::validate\_sample
- is\_user\_defined()
  : stan::gm::function\_signatures
- is\_valid()
  : stan::gm::arg\_adapt\_delta
  , stan::gm::arg\_adapt\_gamma
  , stan::gm::arg\_adapt\_kappa
  , stan::gm::arg\_adapt\_t0
  , stan::gm::arg\_history\_size
  , stan::gm::arg\_id
  , stan::gm::arg\_init\_alpha
  , stan::gm::arg\_int\_time
  , stan::gm::arg\_iter
  , stan::gm::arg\_max\_depth
  , stan::gm::arg\_num\_samples
  , stan::gm::arg\_num\_warmup
  , stan::gm::arg\_refresh
  , stan::gm::arg\_stepsize
  , stan::gm::arg\_stepsize\_jitter
  , stan::gm::arg\_test\_grad\_eps
  , stan::gm::arg\_test\_grad\_err
  , stan::gm::arg\_thin
  , stan::gm::arg\_tolerance
  , stan::gm::singleton\_argument< T >
- is\_void()
  : stan::gm::expr\_type
- iter\_num()
  : stan::optimization::BFGSMinimizer< FunctorType, QNUpdateType, Scalar, DimAtCompile >


---

[ Stan Home Page ]

*© 2011–2014,
Stan Development Team.*
